# Influenza Vaccination in Adults with Chronic Obstructive Pulmonary Disease: The Impact of a Diagnostic Breathing Test on Vaccination Rates

**DOI:** 10.1371/journal.pone.0067600

**Published:** 2013-06-28

**Authors:** Dana S. Mowls, Vinay K. Cheruvu, Melissa D. Zullo

**Affiliations:** University of Melbourne, Australia

## Abstract

**Introduction:**

Influenza vaccination rates are low in adults with chronic obstructive pulmonary disease (COPD). A diagnostic breathing test in adults with COPD may increase vaccination rates; however, research has not demonstrated this relationship. The purpose of this research was to determine if adults with COPD diagnosed by a breathing test were more likely to have had an influenza vaccination during the past 12 months when compared to those with COPD diagnosed without a breathing test.

**Methods:**

This was a cross-sectional study using data from the 2011 Behavioral Risk Factor Surveillance System. Logistic regression examined the relationship between influenza vaccination among adults with COPD diagnosed with a breathing test (n = 13,201) compared to those diagnosed without a breathing test (n = 3,108), after controlling for all potential confounders.

**Results:**

Overall, 49% of respondents with COPD received an influenza vaccination within the past 12 months and 78% reported their COPD was diagnosed by a breathing test. The prevalence of influenza vaccination in the past 12 months was greater in those with COPD diagnosed by a breathing test (53%) compared to those diagnosed without a breathing test (36%). In adjusted analysis, adults with COPD who had a breathing test were 31% (confidence interval 1.1, 1.6) more likely to have received an influenza vaccination in the past 12 months compared to those without a breathing test.

**Discussion:**

A diagnostic breathing test for COPD was associated with increased likelihood of having had an influenza vaccination in the past 12 months. This may be an indicator of the relationship between knowledge of lung function and the need for preventative care, a sign of quality healthcare, or good health-seeking behaviors in patients with COPD. This research is the first to use a nationally representative sample to suggest that spirometry diagnosis of COPD may increase rates of influenza vaccination.

## Introduction

Chronic obstructive pulmonary disease (COPD) is the fourth leading cause of mortality and by 2020 is projected to be the third and fifth leading cause of mortality and disability, respectively [1], [2]. These projections are largely due to acute exacerbations that lead to a rapid decline in physical health [3]. COPD is a leading cause of hospitalization with considerable economic burden [4], [5]. Total United States (US) costs in 2010 were $49.9 billion with direct medical costs (hospital stay, prescription drugs) approximating $29.5 billion [6]. Expenditures for Medicare beneficiaries with COPD are 2.4 times higher compared to those without COPD with exacerbations accounting for 70% of all costs [7]–[9]. Identifying and carrying out methods for preventative care is necessary to reduce the financial and physical burdens of COPD.

Preventative care guidelines for COPD include a yearly influenza vaccination that can reduce the risk of hospitalization, morbidity, and mortality [7], [10]–[13]. The Healthy People 2020 goal for influenza vaccination in adults aged ≥65 and in all high-risk adults is 90% [14]; however, research has consistently shown rates in adults with COPD to be low [15]–[19]. Identifying methods to increase vaccination rates in adults with COPD is important for reducing influenza-related COPD burden. Spirometry, a breathing test to diagnose COPD, may be one method to increase influenza vaccination rates. While spirometry may encourage health care providers to implement interventions including compliance with vaccination recommendations, there is currently no evidence linking spirometry to improvements in influenza vaccination rates [20]. The purpose of this research was to determine if adults with COPD diagnosed by a breathing test were more likely to have had an influenza vaccination during the past 12 months when compared to those with COPD diagnosed without a breathing test. Understanding this relationship may support the use of diagnostic spirometry to impact influenza vaccination rates, reducing the economic and physical burdens of influenza-related COPD exacerbations.

## Methods

### Ethics Statement

As this research involved the analyses of existing data, it was deemed minimal risk and granted exempt status (did not require written informed consent) under federal regulation 45 CFR 46.101(b) by the Institutional Review Board at Kent State University (Protocol #12–559).

### Study Population

Cross-sectional data for the current study were derived from the 2011 Behavioral Risk Factor Surveillance System (BRFSS) [21]. The BRFSS is a federally-funded telephone survey conducted annually by the Centers for Disease Control and Prevention in collaboration with the 50 state health departments and those in Washington, DC; Puerto Rico; the US Virgin Islands; and Guam. The survey uses a multistage cluster design and random digit dialing to select a representative sample of civilian, non-institutionalized adults aged ≥18. In 2011, respondents in all 50 states, Washington, DC, and Puerto Rico, were asked the question “*Has a doctor, nurse, or other health professional EVER told you that you have (COPD) chronic obstructive pulmonary disease, emphysema, or chronic bronchitis?*” In addition to this screening question, optional survey questions related to COPD (COPD module) were administered in 21 states: Arizona; California; Connecticut; Illinois; Iowa; Kansas; Kentucky; Maine; Massachusetts; Michigan; Minnesota; Montana; Nebraska; Nevada; New Jersey; North Carolina; Ohio; Oregon; Tennessee; Utah; West Virginia; and Washington, DC and Puerto Rico. This study was limited to data from these 21 states, Washington, DC, and Puerto Rico (n = 21,567).

### Outcome of Interest: Influenza Vaccination in the Past 12 Months

Influenza vaccination in the past 12 months (yes/no) was assessed by asking the respondent “*During the past 12 months, have you had either a seasonal flu shot or seasonal flu vaccine that was sprayed in your nose?*” Of the 21,567 respondents, 12,303 responded “yes,” 8,926 responded “no,” 58 responded “don’t know/not sure,” and 172 refused to respond.

### Main Exposure of Interest: Breathing Test to Diagnose COPD

Breathing test (yes/no) to diagnose COPD was determined by asking the respondent “*Have you ever been given a breathing test to diagnose your COPD?*” Of the 21,567 respondents, 16,871 responded “yes,” 3,932 responded “no,” 531 responded “don’t know/not sure,” and 233 refused to respond.

### Potential Confounders

To examine the relationship between adults with COPD diagnosed with a breathing test and having received the influenza vaccination within the past 12 months, the following potential confounders were controlled for in analyses: personal doctor (yes/no); health care coverage (yes/no); asthma (yes/no); angina/coronary heart disease/myocardial infarction (yes/no); diabetes (yes/no); smoking (current, former, never); medications taken for COPD (none, one, two, three or more); and demographic variables: gender; age; race/ethnicity (white non-Hispanic, black non-Hispanic, Hispanic, and multi/other); marital status (married, divorced/widowed/separated, and never married/member of an unmarried couple); education (high school graduate/GED or below, and some college/technical school or above); and income (<$15,000, $15,000–$24,999, $25,000–$49,999, and ≥$50,000).

Respondents were excluded who had missing data on the outcome of interest, main exposure, and on any of the potential confounders considered in this study. This resulted in a final sample of 16,309 respondents with complete data who were included in the analysis.

### Statistical Analysis

Sampling weights provided in the 2011 BRFSS public-use data that adjusted for unequal selection probabilities, survey non-response, and oversampling, were used to account for the complex sampling design and to obtain population-based estimates that reflect US non-institutionalized adults.

In order to describe the characteristics of the study population, weighted prevalence estimates, weighted mean estimates, and corresponding 95% confidence intervals (CI), were computed for the outcome of interest, main exposure, and all potential confounders in the study. Student’s t-test for continuous variables and Chi-square test for discrete variables were used to test for significant differences between adults with COPD diagnosed with a breathing test (n = 13,201) when compared to those with COPD diagnosed without a breathing test (n = 3,108). Logistic regression was used to estimate the prevalence odds ratios and 95% CI for receipt of influenza vaccination in the past 12 months associated with breathing test for diagnosis of COPD, controlling for all potential confounders. Analyses were conducted in SAS 9.2 (SAS Institute Inc., Cary, NC, USA) using survey procedures (PROC SURVEYMEANS, PROC SURVEYFREQ, AND PROC SURVEYLOGISTIC) to obtain correct variance estimates, using Taylor series linearization method [22].

## Results

Overall, 49% (CI: 47.6, 51.3) of adults with COPD received an influenza vaccination within the past 12 months (Table 1) and 78% (CI: 75.9, 79.1) reported that their COPD was diagnosed by a breathing test (not shown). The sample was 57% female and 77% white, non-Hispanic. Those with a breathing test when compared to those without a breathing test were more likely to have had an influenza vaccination (53% vs. 36%, respectively; p<0.0001); be older (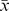 = 58 years vs. 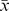 = 51 years, respectively; p<0.0001); be former smokers (39% vs. 29%, respectively; p<0.0001); and have more comorbidity including diabetes (21% vs. 15%, respectively; p<0.001), angina/coronary heart disease/myocardial infarction (27% vs. 14%, respectively; p<0.0001), and asthma (50% vs. 29%, respectively; p<0.0001). Those with a breathing test compared to those without were more likely to report having a personal doctor (92% vs. 81%, respectively; p<0.0001); having health care coverage (89% vs.80%, respectively; p<0.0001); and taking medication for COPD (65% vs. 29%, respectively; p<0.0001). The prevalence of vaccination by age was significantly different between each age category except for between those 65–74 and ≥75 with 22% (CI: 16.2, 27.2), 41% (CI: 37.3, 44.6), 54% (CI: 50.8, 56.8), 65% (CI: 62.3, 67.8), and 68% (CI: 64.9, 71.1) vaccinated in the age ranges <40, 40–54, 55–64, 65–74, and ≥75 years, respectively (data not shown).

**Table 1:** Characteristics of respondents with COPD: frequency, weighted prevalence, and 95% confidence intervals, overall and by breathing test to diagnose COPD, 2011 BRFSS, n = 16,309.

	Overall		Breathing Test		No Breathing Test		
	(n = 16,309)		(n = 13,201)		(n = 3,108)		
	N (%)	(95% CI )	N (%)	(95% CI )	N (%)	(95% CI )	*P*
**Ever had a seasonal flu shot or spray?** (Yes)	9403 (49.4)	(47.6–51.3)	7981 (53.3)	(51.2–55.4)	1422 (36.0)	(32.4–39.7)	<0.0001
**Age**, mean (SD)	56.5 (0.4)	(55.8–57.2)	58.1	(57.3–58.9)	51.0	(49.3–52.6)	<0.0001
**Age**							<0.0001
<40 years old	816 (14.9)	(12.9–16.9)	538 (12.0)	(9.9–14.1)	278 (25.0)	(20.3–29.6)	
40 to 54 years old	3129 (28.4)	(26.7–30.2)	2318 (27.0)	(25.0–29.0)	811 (33.3)	(29.7–37.0)	
55 to 64 years old	4638 (23.9)	(22.6–25.3)	3702 (25.3)	(23.7–26.9)	936 (19.2)	(16.8–21.6)	
65 to 74 years old	4483 (19.2)	(18.0–20.3)	3867 (21.0)	(19.7–22.4)	616 (12.8)	(10.5–15.2)	
≥75 years old	3243 (13.5)	(12.6–14.4)	2776 (14.7)	(13.6–15.7)	467 (9.6)	(7.9–11.4)	
**Gender**							0.97
Male	5589 (42.7)	(40.8–44.6)	4595 (42.7)	(40.5–44.8)	994 (42.9)	(38.7–47.1)	
Female	10720 (57.3)	(55.4–59.2)	8606 (57.3)	(55.2–59.5)	2114 (57.1)	(52.9–61.3)	
**Race/Ethnicity**							<0.0001
White non-Hispanic	13628 (77.0)	(75.1–78.9)	11030 (77.8)	(75.7–79.9)	2598 (74.5)	(70.3–78.7)	
Black non-Hispanic	1091(8.7)	(7.3–10.1)	910 (9.5)	(7.8–11.2)	181 (6.0)	(4.2–7.7)	
Hispanic	684 (7.9)	(6.6–9.1)	529 (6.0)	(4.9–7.0)	155 (15.0)	(10.5–18.5)	
Other/multiracial, non-Hispanic	906 (6.4)	(5.3–7.5)	732 (6.8)	(5.4–8.1)	174 (5.0)	(3.2–6.9)	
**Education Level**							0.24
High school graduate or below	8252 (53.7)	(52.1–55.8)	6681 (54.3)	(52.2–56.3)	1571 (51.6)	(47.5–55.7)	
Some college, technical school or college graduate	8057 (46.3)	(44.2–47.8)	6520 (45.7)	(43.7–47.8)	1537 (48.4)	(44.3–52.5)	
**Income Level**							0.25
<$15,000	4112 (24.9)	(23.3–26.5)	3369 (25.1)	(23.3–26.9)	743 (24.5)	(21.0–27.9)	
$15,000–$24,999	4487 (23.9)	(22.5–25.4)	3658(23.8)	(22.3–25.4)	829 (24.3)	(20.9–27.8)	
$25,000–$49,999	4504 (27.5)	(25.9–29.1)	3654 (28.2)	(26.4–30.0)	850 (25.0)	(21.8–28.2)	
≥$50,000	3206 (23.6)	(21.9–25.4)	2520 (22.9)	(20.9–24.9)	686 (26.2)	(22.3–30.2)	
**Marital Status**							0.001
Married/member of unmarried couple	6726 (46.9)	(45.1–48.8)	5394 (46.7)	(44.8–48.9)	1332 (47.1)	(43.0–51.2)	
Divorced/separated/widowed	8027 (37.9)	(36.2–39.6)	6614 (39.5)	(37.4–41.2)	1413 (33.0)	(29.6–36.5)	
Never married	1556 (15.2)	(13.5–16.9)	1193 (13.8)	(12.0–15.6)	363 (19.9)	(15.6–24.2)	
**Smoking Status**							<0.0001
Current smoker	5658 (38.9)	(37.1–40.8)	4306 (38.5)	(36.4–40.6)	1352 (40.4)	(36.5–44.3)	
Former smoker	7043 (36.7)	(35.0–38.3)	6117(39.0)	(37.1–40.9)	926 (28.6)	(25.0–32.3)	
Never smoker	3608 (24.4)	(22.6–26.1)	2778 (22.5)	(20.6–24.3)	830 (31.0)	(26.9–35.0)	
**Ever told you had diabetes?** (Yes)	3666 (19.1)	(17.9–20.4)	3308 (20.7)	(18.9–21.8)	535 (14.9)	(12.3–17.4)	<0.001
**Ever told you had angina, CHD or MI?** (Yes)	4226 (23.9)	(22.4–25.4)	3741 (26.9)	(25.1–28.7)	485 (13.7)	(11.2–16.1)	<0.0001
**Ever told you had asthma?** (Yes)	7080 (45.6)	(43.7–47.4)	6293 (50.3)	(48.2–52.3)	787 (29.4)	(25.2–33.6)	<0.0001
**Have a personal doctor?** (Yes)	15191 (89.6)	(88.1–91.1)	12490 (92.2)	(90.7–93.7)	2701 (80.7)	(76.8–84.6)	<0.0001
**Medications for COPD**							
None	6344 (42.9)	(41.1–44.8)	4228 (34.7)	(32.7–36.8)	2116 (71.2)	(67.6–74.8)	<0.0001
One	3544 (21.4)	(19.9–22.8)	3011 (22.6)	(20.9–24.2)	533 (17.3)	(14.1–20.5)	
Two	3012 (16.9)	(15.5–18.2)	2742 (19.7)	(18.1–21.4)	270 (7.0)	(5.1–8.8)	
Three or more	3409 (18.8)	(17.5–20.1)	3220 (23.0)	(21.3–24.6)	189 (4.5)	(3.3–5.8)	
**Health Care Coverage** (Yes)	14883 (87.0)	(85.5–88.5)	12213 (89.2)	(87.6–90.8)	2670 (79.6)	(75.9–83.3)	<0.0001

COPD = chronic obstructive pulmonary disease; BRFSS = Behavioral Risk Factor Surveillance System; SD = standard deviation; CHD = coronary heart disease; MI = myocardial infarction.

In unadjusted analyses, respondents with a breathing test were 2.0 times (CI: 1.7, 2.4) more likely to have received the influenza vaccination in the past 12 months when compared to those without a breathing test (Table 2). All variables in the unadjusted models were significant with the exception of gender, education level, and asthma. In adjusted analysis, those with a breathing test were 31% (CI: 1.1, 1.6) more likely to have received the influenza vaccination in the past 12 months when compared to those without a breathing test when controlling for age; race/ethnicity; income level; marital status; smoking status; diabetes; angina/coronary heart disease/myocardial infarction; personal doctor; medications for COPD; and health care coverage. There was a significant trend (p<0.001) for odds of vaccination with increasing categories of age.

**Table 2:** Unadjusted and adjusted weighted prevalence odds ratios and 95% confidence intervals for ever had a seasonal flu vaccination among persons with COPD in the 2011 BRFSS, n = 16,309.

	UOR (95% CI)	*P*	AOR (95% CI )
Breathing test to diagnose COPD? (Yes)	2.03 (1.70–2.43)	<0.0001	1.31 (1.08–1.60)
**Age**		<0.0001	
<40 years old	Reference		Reference
40 to 54 years old	2.50 (1.75–3.56)		1.80 (1.26–2.58)
55 to 64 years old	4.21 (2.99–5.92)		2.63 (1.86–3.72)
65 to 74 years old	6.71 (4.76–9.45)		3.45 (2.44–4.88)
≥75 years old	7.67 (5.40–10.88)		3.73 (2.61–5.34)
**Gender**		0.09	
Male	Reference		–
Female	1.15 (0.98–1.34)		–
**Race/Ethnicity**		<0.0001	
Black non-Hispanic	Reference		Reference
White non-Hispanic	2.64 (1.93–3.61)		2.53 (1.86–3.44)
Hispanic	1.53 (0.98–2.41)		1.99 (1.27–3.13)
Other/multiracial, non-Hispanic	2.14 (1.33–3.45)		2.30 (1.43–3.69)
**Education Level**		0.84	
Some college, technical school or college graduate	Reference		–
High school graduate or below	1.02 (0.88–1.18)		–
**Income Level**		<0.001	
<$15,000	Reference		Reference
$15,000–$24,999	1.46 (1.20–1.78)		1.35 (1.08–1.69)
$25,000–$49,999	1.45 (1.18–1.77)		1.23 (0.98–1.54)
≥$50,000	1.24 (0.98–1.56)		1.23 (0.96–1.58)
**Marital Status**		<0.0001	
Never married	Reference		Reference
Married/member of unmarried couple	1.75 (1.34–2.29)		0.82 (0.61–1.10)
Divorced/separated/widowed	1.95 (1.49–2.54)		0.85 (0.63–1.15)
**Smoking Status**		<0.0001	
Current smoker	Reference		Reference
Former smoker	2.12 (1.81–2.50)		1.30 (1.10–1.55)
Never smoker	1.25 (1.01–1.53)		1.25 (1.02–1.54)
**Ever told you had diabetes?** (Yes)	1.82 (1.54–2.14)	<0.0001	1.43 (1.20–1.71)
**Ever told you had angina, CHD, or MI?** (Yes)	1.46 (1.23–1.74)	<0.0001	0.93 (0.79–1.10)
**Ever told you had asthma?** (Yes)	1.08 (0.93–1.25)	0.34	–
**Have a personal doctor?** (Yes)	5.77 (3.96–8.39)	<0.0001	2.37 (1.63–3.44)
**Medications for COPD**		<0.0001	
None	Reference		Reference
One	1.57 (1.30–1.91)		1.32 (1.08–1.61)
Two	2.19 (1.77–2.73)		1.71 (1.38–2.12)
Three or more	2.70 (2.20–3.31)		2.02 (1.64–2.50)
**Health Care Coverage** (Yes)	4.48 (3.31–6.07)	<0.0001	2.28 (1.68–3.10)

COPD = Chronic Obstructive Pulmonary Disease; BRFSS = Behavioral Risk Factor Surveillance System; UOR = unadjusted odds ratio; AOR = adjusted odds ratio; CHD = coronary heart disease; MI = myocardial infarction.

Respondents excluded from analyses (n = 5,258) compared to those included (n = 16,309) were more likely to be female (62% vs. 57%, respectively; p = 0.03); be never married (19% vs. 15%, respectively; p = 0.02); be high school graduates/below (65% vs. 54%, respectively; p<0.0001 ); have an income ≤$24,999 (57% vs. 49%, respectively; p = 0.01); not have a personal doctor (15% vs. 10%, respectively; p = 0.01); have diabetes (23% vs. 19%, respectively; p = 0.01); and not take medications for COPD (49% vs. 43%, respectively; p = 0.01).

## Discussion

The prevalence of having an influenza vaccination in the past 12 months was greater in those with COPD diagnosed by a breathing test (53%) compared to those diagnosed without a breathing test (36%). More importantly, a significant association was observed between having had a breathing test to diagnose COPD and having had the influenza vaccination, with those who had a diagnostic breathing test being 31% more likely to have received an influenza vaccination in the past 12 months compared to those who had not been diagnosed by a breathing test. This research is the first in a nationally representative sample to demonstrate that COPD diagnosis by a breathing test is associated with receiving the influenza vaccine and suggests that spirometry may be a method to increase rates of vaccination.

Influenza vaccination may result in declines in mortality, morbidity, and health care utilization in adults with COPD and guidelines recommend annual vaccination in this high-risk group [7], [10], [13]. The Healthy People 2020 national vaccination target for both high-risk individuals and those ≥65 years is 90% [14]. Therefore, it is particularly problematic that in the current research the overall prevalence of having received the influenza vaccination in the past 12 months did not approach this target. This finding is supported by previous research showing low (40%) vaccination in hospitalized COPD patients [17]. Research demonstrating national rates have shown that only 19% of adults age 18–49 and 44% of adults age 50–64 with chronic lung diseases had the influenza vaccine [19]. More recent national research showed a crude prevalence of 43% and differences in adjusted prevalence by age and race in those with COPD age 18–64 (31% in whites and 21% in blacks; p = 0.048) and in those ≥65 (76% in whites and 63% in blacks; p = 0.15) [15]. Estimates of high-risk groups show low vaccination rates that differ by age and that are below recommendations even in the oldest ages [16], [18], [19]. Low vaccination rates may be associated with preventable exacerbations. Influenza is estimated to cause 8–36% of exacerbations, with higher incidence during epidemics, and results in greater health care utilization in high-risk compared to healthy elderly who are more likely to have a doctor visit (60% compared to 42%, respectively) and to require hospitalization (20% compared to none, respectively) [13], [23]–[27]. While influenza vaccination has been shown to reduce exacerbations [13], there is mixed-evidence on its ability to reduce hospitalization and mortality in COPD specifically, but some evidence in combined chronic lung diseases. While Nicol found a 52% reduction in hospitalization for influenza and pneumonia and a 70% reduction in risk of death from any cause in vaccinated compared to unvaccinated older adults, these findings were in chronic lung diseases, not specifically COPD [28], [29]. Hospitalization and mechanical ventilator use in vaccinated patients has been shown to be lower than when compared to unvaccinated adults; however, the sample size may have been too small to determine a difference due to low influenza incidence [13]. Poole found that vaccination did not reduce hospitalization or mortality in adults with COPD [30]. Finally, a meta-analysis of observational studies showed vaccination reduced hospitalizations by 50% in older adults with COPD [31]; although, many of these studies had higher influenza incidence as they were conducted during epidemics and this may have contributed to the significant findings. Despite these mixed-results, influenza vaccination is cost-effective with estimated net savings per year/per vaccinated high-risk person being at least $171 [28]. Even with vaccination recommendations, and the clinical and economic benefit, vaccination does not approach recommended levels. Increasing vaccination rates requires overcoming established barriers to vaccination including perception of low risk, fear of side effects, fear of contracting influenza from the vaccine, and perceived need for vaccination [32]. In adults with COPD, there is the repudiated concern among healthcare professionals and patients that vaccination may be associated with increased risk for exacerbation prior to immunity development [17]. While the current research shows that a diagnostic breathing test is associated with improved vaccination rates, this intervention alone is not enough to approach 90% and overcoming known barriers to vaccination is an important component to increasing vaccination rates in adults with COPD.

Multiple reports address the lack of evidence between spirometry and disease outcomes or treatment in adults with COPD and an important question is whether spirometry contributes to increased influenza vaccination [20], [33]. The current research observed that it is associated with influenza vaccination. Therefore, it is unfortunate that given the relationship between a diagnostic breathing test and vaccination that research conducted in the US has shown that only 33% of adults with COPD have had diagnostic spirometry and use decreases in older adults with 32%, 28%, and 15% of older adults aged 65–74, 75–84, and ≥85, respectively, having had diagnostic spirometry [34], [35]. These figures are discouraging as older adults are at highest risk for flu-related hospitalization and mortality [36] and if spirometry can increase vaccination rates in high-risk groups, it is important to increase spirometry as one inexpensive intervention to increase vaccination. In the current research the prevalence of influenza vaccination increased with age (not shown). When compared to individuals less than 40 years, individuals ≥40 classified by age group had increasing and significant odds of influenza vaccination (trend p<0.001). Interestingly, there were differences in breathing test prevalence between each age group indicating that it may not be just increasing age, as demonstrated by previous research showing older adults have higher prevalence of vaccination, that contributes to the high odds of vaccination but also the use of a breathing test; however, this could not be examined in the current research and there may be other factors that need to be considered in future research. There were significant differences in medication use among those with and without a diagnostic breathing test. Adults with a breathing test were more likely to use one or more medications for COPD. Medication use in COPD presumably serves as a proxy for disease severity and as such the assumption can be made that disease severity contributes to the increased likelihood of vaccination. Finally, previous research on spirometry prevalence are in marked contrast to the 78% of respondents in the current, nationally representative sample who indicated they have had a breathing test to diagnose COPD; yet, this finding is consistent with a recent Centers for Disease Control and Prevention report utilizing 2011 BRFSS data that found 76% of respondents with COPD had been given a diagnostic breathing test and that breathing test diagnosis increased with age [37]. The reasons for this higher prevalence of a breathing test deserve further investigation. Possible explanations include: previous research largely comes from smaller clinical studies; because 46% of respondents in the current research also had asthma and may have had a diagnostic breathing test for this and thought it was for COPD (however, removing those with asthma did not change the prevalence; data not shown); due to overestimation from self-report; or because “diagnosis by a breathing test” is not diagnosis by spirometry; however, this last reason is unlikely to be the cause of the higher prevalence because spirometry is the main test to diagnose COPD and respondents are undoubtedly more likely to know they were diagnosed by a breathing test than to remember the clinical terminology: “spirometry.” Regardless, the high diagnostic breathing test prevalence is promising to the field of pulmonary medicine and supports the suggestion that it may not be just increasing age that encourages a vaccine but also spirometry.

Potential explanations, limitations, and strengths of the current research include that findings may be due to differences in health seeking behaviors, healthcare access, and physician factors that could not be examined in this research. Research by Zimmerman described factors that seem to support this idea including that persons who are more likely to receive vaccination include those who know that Medicare covers vaccination costs, the belief that the doctor recommends the vaccine, the belief that it is important, and receiving care at the Veterans Affairs [32]. Further, having evidence of lung function may encourage clinicians and patients to engage in preventative efforts they would not otherwise do, if they did not have documentation of lung function. These potential explanations should be examined in future research. Limitations include that temporality or length of time between the breathing test and influenza vaccination could not be established, and a breathing test to diagnose COPD may not be the same as clinically administered spirometry, as discussed. In addition, individuals in long-term care facilities were not sampled and this may impact rates of vaccination as these groups tend to be older and sicker. Finally, severity of flu season can vary from year to year and this may influence an individual’s perceptions of need for vaccination. This may be evidenced by the different rates of vaccination found in the literature and discussed above. However, the Healthy People 2020 recommended target for vaccination is 90% in adults with chronic conditions and this recommendation does not vary from year to year. In light of the newness of the BRFSS COPD module, analysis of additional years of data may demonstrate differing rates of vaccination associated with an epidemic. This is an area for future research. Strengths of this research include that it is from a nationally representative sample and is the first to demonstrate that a breathing test to diagnose COPD is associated with having had a flu vaccine in the previous 12 months.

Confirmation of COPD with spirometry is an objective way to detect symptomatic COPD patients and the associated benefits of spirometry have yet to be fully understood. This research provides evidence that a breathing test is a critical component of care associated with increased vaccination rates and therefore can impact influenza-related exacerbations. Every effort should be made to ensure adults with COPD receive the influenza vaccine.
