# Correction: Influenza Vaccination in Adults with Chronic Obstructive Pulmonary Disease: The Impact of a Diagnostic Breathing Test on Vaccination Rates

**DOI:** 10.1371/annotation/c472fa25-5876-4b8e-bb3d-4f23e68bb277

**Published:** 2013-07-18

**Authors:** Dana S. Mowls, Vinay K. Cheruvu, Melissa D. Zullo

None of the authors' affiliations were indicated. All authors are affiliated with: 1. College of Public Health, Kent State University, Kent, Ohio, United States.

